# Obituary (1923–2023): In Memoriam Álvaro Guimarães e Sousa

**DOI:** 10.1007/s00266-023-03355-6

**Published:** 2023-05-01

**Authors:** José Manuel Amarante

**Affiliations:** Universidade do Porto and Cooperativa de Ensino Superior Universitário e Politécnico, Porto, Portugal

*No Level Assigned* This journal requires that authors assign a level of evidence to each submission to which Evidence-Based Medicine rankings are applicable. This excludes Review Articles, Book Reviews, and manuscripts that concern Basic Science, Animal Studies, Cadaver Studies, and Experimental Studies. For a full description of these Evidence-Based Medicine ratings, please refer to the Table of Contents or the online Instructions to Authors www.springer.com/00266

Álvaro Guimarães e Sousa (1923–2023) was a precursor of the practice of Plastic Surgery in Portugal to whom the specialty owed much, died on February 20, 2023
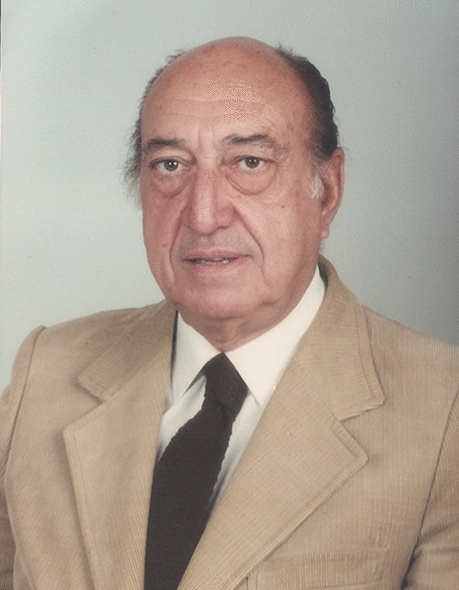


Graduated from the Faculty of Medicine of Porto in 1950, managing to overcome a difficult personal moment.

Then he began training in General Surgery at the General Hospital of Santo António in Porto for 5 years. Motivated by reading an article reporting the reconstruction of a lower limb by an English plastic surgeon, he decided in 1955 to leave for England to specialize in Plastic Surgery.

He had as a reference in London Ruela Ramos, the first anaesthesiologist of Porto, who knew well the hospital where both worked. As soon as London arrived, on a cold and rainy January night, he soon sought him out after the long journey by car.

Two days later, recovered from the gruelling journey, he was already working at Oxford in the Nuffeld Department of Plastic Surgery at Churchill Hospital, a service run by Tomas Pomfret Kilner (1890–1964). At the time, he was the only professor of plastic surgery in the UK who, like Gillies, had gained experience in World War I, treating pilots burned and wounded in combat.

Sometime later, on Kilner's advice, he chose to continue training in Bristol as Senior House Officer in the Department of Plastic and Jaw Surgery at Frenchay Hospital, where he spent three years.

After finishing his training in 1959, he was invited to return again to Oxford, to Churchill Hospital, now as Senior Registrar, a position he occupied for a short time since, for imperious family reasons, he had to return to Porto.

However, a year before he returned, in 1959, during the holidays, he had requested the Northern Regional Council of the Medical Association for the recognition of the specialty acquired in England and that in Portugal did not exist. For seven years, he fought for Plastic Surgery to be considered an autonomous specialty of General Surgery at a time when, in the country, the vast majority of surgeons, the Medical Association and even some Faculties of Medicine were unaware of the specialty and its scope of action.

As a result of his diligence and insistence, Dec. 1059 of December 2, 1964, is published by the Ministry of Corporations and Social Security—based on the favourable opinion of the National Board of Education, after hearing three professors from each of the Faculties of Medicine (one voted against)—authorizing the Medical Association to create the specialty.

Thus was born on November 4, 1966, the new specialty—Plastic and Reconstructive Surgery.

Very close to the end of his career, in 1991, he decided to leave the city of Porto and began to live on his property, in the rural environment of the Lima River valley, having also worked for two years in the hospital of Viana de Castelo.

Since his return from England, he has always practiced public and private practice, namely at the Casa de Saúde da Boavista.

In 1993, all clinical activity came to an end.

Guimarães e Sousa was undoubtedly the great driver of Reconstructive Plastic Surgery, having played a relevant role in the beginning and evolution of the specialty in Portugal.

He trained twelve surgeons of great prominence, five of whom were later appointed directors of Plastic and Reconstructive Surgery services in various hospitals.

Álvaro Guimarães e Sousa had a strong physical presence, which intimidated; however, he was an affable, interested and cultured colleague, clearly denoting in the conviviality that England had marked him a lot beyond the training in surgery.

He had a long life lived lucidly and autonomously until a few days before he died on his property in the north of the country, in the region of Viana do Castelo.

